# Supplementary material for: Lumbar spine MRI annotation with intervertebral disc height and Pfirrmann grade predictions
Source: PLoS One. 2024 May 10;19(5):e0302067. doi: 10.1371/journal.pone.0302067 (PMC11086861; doi:10.1371/journal.pone.0302067)
Supplement: Attachment — Submitted filename: Response to Reviewers.docx [file pone.0302067.s001.docx]

Response to Reviewers’ Feedback

Paper Title:	Lumbar spine MRI annotation with intervertebral disc height and Pfirrmann grade predictions

Paper ID#:	PONE-D-24-05241.

Reviewers’ and Editor’s comments are in black. Our response are in red.

----

When submitting your revision, we need you to address these additional requirements.

1. Please ensure that your manuscript meets PLOS ONE's style requirements, including those for file naming. The PLOS ONE style templates can be found at https://journals.plos.org/plosone/s/file?id=wjVg/PLOSOne_formatting_sample_main_body.pdf and https://journals.plos.org/plosone/s/file?id=ba62/PLOSOne_formatting_sample_title_authors_affiliations.pdf

**Our response:**

We have reviewed the manuscript again and have made the following changes based on the templates.

- Moved the title down a few lines, remove the bold font style, and reduce the font size to 14.
- We have also changed the way the headings are written to ensure that only the first word is capitalized.

2. Thank you for stating the following in the Acknowledgments Section of your manuscript:

“This work is supported, in part, by the Ministry of Education, Culture, Research, and Technology of the Republic of Indonesia under grant number: 004-RD-LPPM-UMN/ P-HD/VI/2022.”

We note that you have provided funding information that is currently declared in your Funding Statement. However, funding information should not appear in the Acknowledgments section or other areas of your manuscript. We will only publish funding information present in the Funding Statement section of the online submission form.

Please remove any funding-related text from the manuscript and let us know how you would like to update your Funding Statement. Currently, your Funding Statement reads as follows:

“Initials of the authors who received each award: FN

Grant numbers awarded to FN: 004-RD-LPPM-UMN/ P-HD/VI/2022.

The full name of funder: the Ministry of Education, Culture, Research, and Technology of the Republic of Indonesia

URL of funder website: https://www.kemdikbud.go.id/

Did the sponsor or funder play any role in the study design, data collection and analysis, decision to publish, or preparation of the manuscript? No”

Please include your amended statements within your cover letter; we will change the online submission form on your behalf.

**Our response:**

We have removed the Acknowledgement section from the manuscript. The Funding Statement in the online submission form will remain the same.

3. When completing the data availability statement of the submission form, you indicated that you will make your data available on acceptance. We strongly recommend all authors decide on a data sharing plan before acceptance, as the process can be lengthy and hold up publication timelines. Please note that, though access restrictions are acceptable now, your entire data will need to be made freely accessible if your manuscript is accepted for publication. This policy applies to all data except where public deposition would breach compliance with the protocol approved by your research ethics board. If you are unable to adhere to our open data policy, please kindly revise your statement to explain your reasoning and we will seek the editor's input on an exemption. Please be assured that, once you have provided your new statement, the assessment of your exemption will not hold up the peer review process.

**Our response:**

In the last round of the peer-review process, we have already provided a publicly-shared folder on OneDrive where the reviewers can investigate the data that we used. The URL was <https://bit.ly/PLOSPFG2024>

We have now moved the data to Mendeley Data and can be accessed freely via the following URL: <https://data.mendeley.com/datasets/x6ggzp2ycn/1>

4. Please review your reference list to ensure that it is complete and correct. If you have cited papers that have been retracted, please include the rationale for doing so in the manuscript text, or remove these references and replace them with relevant current references. Any changes to the reference list should be mentioned in the rebuttal letter that accompanies your revised manuscript. If you need to cite a retracted article, indicate the article’s retracted status in the References list and also include a citation and full reference for the retraction notice.

**Our response:**

We have checked the references to ensure that they are correctly cited and none of them had been retracted. We made the following changes:

- Corrected reference [1] and [26] items which were incorrectly referred to as [Internet] because the reference manager has a URL in their record.
- Updated the URL of reference [4] because the old one has changed.
- Updated the last accessed date of all web page reference items [3, 4, 10, 17] to 25 March 2024
- Replaced reference item [30] with a new one due to the article’s recent retraction.

The previous one was:

30. Chugh H, Gupta S, Garg M, Gupta D, Juneja S, Turabieh H, Na Y, Kiros Bitsue Z, Others. Image retrieval using different distance methods and color difference histogram descriptor for human healthcare. J Healthc Eng. 2022;2022.

The new one is:

30. Hee-Hyung BuNam-Chul Kim Byoung-Ju Yun, Kim S-H. Content-Based Image Retrieval Using Multi-Resolution Multi-Direction Filtering-Based CLBP Texture Features and Color Autocorrelogram Features. J Inf Process Syst. 2020;16(4):991–1000.

This change does not change or invalidate the statement in the manuscript in which the article was referenced. It was used to back up the argument that states “The majority of information in a color correlogram is concentrated along the main diagonal cells of the matrix hence several studies have suggested not to use the entire matrix but only these cells [30,31]”. The replacement article also supports the same argument.

Additional Editor Comments:

The manuscript is well written and basically acceptable after minor revision.

Comments:

1. The manuscript should contain an Introduction, Materials and Methods, Results, Discussion, and Conclusion section. Please revise the manuscript.

2. The literature review section should be combined with the introduction section. These two sections are too long. Please summarize the content and shorten the descriptions.

**Our response:**

Thank you for the comments. We have reviewed the structure of our manuscript and made the following changes:

- Combined the Introduction section and the Literature Review section into one section, and shorten the result by ~20%.
- Replaced the title of the Methodology section to Materials and Methods, and
- Replaced the title of the Experimental results and analysis section to Results and discussion.

Reviewers' comments:

Reviewer's Responses to Questions

Comments to the Author

1. Is the manuscript technically sound, and do the data support the conclusions?

The manuscript must describe a technically sound piece of scientific research with data that supports the conclusions. Experiments must have been conducted rigorously, with appropriate controls, replication, and sample sizes. The conclusions must be drawn appropriately based on the data presented.

Reviewer #1: Yes

Reviewer #2: Yes

________________________________________

2. Has the statistical analysis been performed appropriately and rigorously?

Reviewer #1: Yes

Reviewer #2: Yes

________________________________________

3. Have the authors made all data underlying the findings in their manuscript fully available?

The PLOS Data policy requires authors to make all data underlying the findings described in their manuscript fully available without restriction, with rare exception (please refer to the Data Availability Statement in the manuscript PDF file). The data should be provided as part of the manuscript or its supporting information, or deposited to a public repository. For example, in addition to summary statistics, the data points behind means, medians and variance measures should be available. If there are restrictions on publicly sharing data—e.g. participant privacy or use of data from a third party—those must be specified.

Reviewer #1: Yes

Reviewer #2: Yes

________________________________________

4. Is the manuscript presented in an intelligible fashion and written in standard English?

PLOS ONE does not copyedit accepted manuscripts, so the language in submitted articles must be clear, correct, and unambiguous. Any typographical or grammatical errors should be corrected at revision, so please note any specific errors here.

Reviewer #1: Yes

Reviewer #2: Yes

________________________________________

5. Review Comments to the Author

Please use the space provided to explain your answers to the questions above. You may also include additional comments for the author, including concerns about dual publication, research ethics, or publication ethics. (Please upload your review as an attachment if it exceeds 20,000 characters)

Reviewer #1: We believe this study is useful in the context of the need to rationalize the need for new approaches to increase the efficiency and effectiveness of the radiological diagnostic process. I would like you to continue your research for more accuracy.

Reviewer #2: The authors reported a fascinating application of deep learning in lumbar MRI analysis. By training a deep learning model on a large dataset of annotated MRI images with corresponding Pfirrmann grades, they developed a system that can automatically evaluate and grade intervertebral discs based on their appearance in the images.

As the author pointed out in the discussion section, misclassification of 23.8% of IVDs that have Pfirrmann Grade 5 was a concern of this study (Misclassification of 30% of Pfirrmann Grade 1 IVDs as having Pfirrmann Grade 2 was not a problem because IVDs with both of Pfirrmann Grade 1 and 2 are healthy). Calculating the intervertebral disc height at three points (posterior, anterior, and midline) like "disc height index" might indeed provide a more comprehensive evaluation of disc morphology and potentially enhance the accuracy of the disc height assessment.

I hope that this system will make steady progress and potentially assist radiologists in their diagnosis and treatment planning processes, as well as enable faster and more consistent evaluations across different healthcare settings.

**Our response:**

Thank you for your time and effort to review our paper. We are glad that both of you found our work to be useful and fascinating. The team hopes that we can build on this work and implement the solution more widely and increase its positive impact to society.
